# Microscopical Studies upon the Absorption of the Roots of Temporary Teeth

**Published:** 1885-02

**Authors:** Frank Abbott


### Microscopical Studies upon the Absorption of the Roots of Temporary Teeth.

BY FRANK ABBOTT, M. D.

Whitney Memorial Prize Essay of the Dental Society of the State of New York.

The fact that temporary teeth, previous to the appearance of the permanent ones, are considerably reduced in size, often lacking roots, often consisting only of a thin shell, and exhibiting a corroded, festooned surface, has long since attracted the attention of observers. It has never been doubted that a persistent though gradual irritation causes the absorption; but what the real cause of this irritation is, we can not tell. The idea that in all cases the pressure of the growing permanent tooth is the direct cause must be abandoned, since clinical observation shows that the absorption of a temporary tooth may take place though far distant from the permanent one; nevertheless, we maintain that the growth of the latter, indirectly at least, causes the irritation, and consequent absorption.

The assertion of Tomes that it is due to the presence of freely vascularized papilla does not explain the decrease of the dental tissues, for the papilla is nothing but medullary tissue, such as we meet with in any part of the organism where one tissue is about to change into another. Such a papilla can be the cause of the absorption, as well as its result. Another assertion, that the medullary cells eat out the dental tissues by their active growth, or by their amœboid motions, is insufficient for the explanation of the loss of the lime-salts in the dental tissue, the presence of circular or semi-circular excavations and bays, so characteristic of the melting process of the cementum and dentine of diciduous teeth.

Since we know that pieces of dead bone or ivory may be absorbed with figures similar to those found on the surface of temporary teeth, the idea possibly becomes admissable that, owing to the presence of an acid, first the lime-salts are dissolved out within certain territories of the dead bone tissue in a merely chemical or passive way, whereupon the soft medullary tissue penetrates the space thus established. Quite different, however, will be the conception of this process if we bear in mind that the temporary teeth, as well as the permanent ones, are made up of living tissues, and an active participation of these tissues must be expected in the process of transformation of the dental into that of medullary tissue. As the process of absorption is closely allied to the process of inflammation, and active changes of the dental tissues have been proven to follow inflammation beyond any doubt, we may *a priori* expect such changes of the bone tissues of the temporary teeth in the process of absorption also. I shall try to prove in this paper that such changes really do occur. In the light of the most advanced modern views concerning the structure of the dental tissues, we consider cementum, dentine, and enamel as endowed with properties of life, or, in other words, as pervaded by living matter in the shape of an extremely delicate reticulum. In this view, not only the cement corpuscles and their coarser offshoots contain living matter in the shape of so-called "granular protoplasm," but the whole basis-substance present between the cement corpuscles is alive also, only the minute meshes of the living reticulum holding a gluey basis-substance saturated with lime-salts. In the dentine, not only the tenants of the dentinal canaliculi (the dentinal fibres) are alive, but the whole mass of gluey and calcified basis-substance between the dentinal canaliculi is also living. The same holds good for the enamel, in which the delicate fibrilæ between the enamel prisms have positively been proven to be living matter, but the prisms are pierced by living matter, though the latter has not been demonstrated directly, but indirectly in morbid changes of the enamel. Of the cementum, we know that each cement corpuscle occupies the center of a more or less globular territory of basis-substance. If, therefore, circular fields of absorption appear in the process of inflammation and absorption of the cementum, we can readily trace these territories in following out the portion affected by the process of absorption. But how shall we explain the bay-like excavations in the dentine and enamel, so often seen in reduced temporary teeth where there is nothing known of territories? Here the first difficulty sets in, due to the lack of knowledge of the history of the development of dentine and enamel. Czermak's inter-globular spaces indicate the presence of such territories in the dentine, the presence of which, however, can be proven only after accurate researches in the history of development.

Granted that the dissolution of lime-salts takes place in globular territories in the dental tissues, the next question will be, how do the medullary elements appear in such spaces? Do they migrate or penetrate from without, or do they originate, in part at least, from the living material present in all dental tissue?

### ABSORPTION OF CEMENTUM.

The process of absorption of a provisional tooth begins on the cementum of the roots. The latter exhibits, before the beginning of this process, the features of cementum of permanent teeth. Primarily the absorption is marked by the appearance of the well-known fields so commonly met with in the process of ostitis, that is, excavations on the surface, either semi-circular or composed of a varying number of semi-circular festoons, all of which are filled with medullary elements, multinuclear bodies, or a delicate myxomata, in part a bony, in part fibrous connective tissue, blending with the adjacent myxomatous or fibrous pericementum. The communication of the excavations with the pericementum is either widely gaping or through a narrowed neck on the surface of the cementum. Sometimes, however, in the sections, the excavations appear isolated, without any communication with the surface, which latter instance, however, will certainly not entitle us to deny the existence of such a communication on a plane above or below that of the section. In the excavations the cementum is unquestionably reduced, first into medullary, afterward into myxomatous or fibrous tissue. By closely watching excavations, of a more recent date, at the periphery of those in communication with the pericementum, we notice that the lime-salts and the basis-substance proper are missing, and are replaced by a uniformly granular protoplasm, or a varying number of faintly marked medullary elements, each of which may contain a central nucleus. We can trace a gradual change of the tissue of cementum from a dissolution of lime-salts to the appearance of a mass of granular protoplasm, and at last to the formation of medullary corpuscles. The circular shape of the excavation is in all cases undoubtedly due to a dissolution of the lime-salts, and afterwards to the basis-substance proper, within the territory of of a cement corpuscle. Sometimes we see an enlargement of the lacuna and the cement corpuscle itself, the latter splitting up into a varying number of glistening lumps, which are readily stained by an ammoniacal solution of carmine. In other instances the whole territory of a cement corpuscle is transformed into protoplasm, and the reappearance of such protoplasm is traceable through broad offshoots to neighboring cement corpuscles. In a third instance a varying amount of territory has assumed a delicate fibrous appearance, caused by an early grouping of the medullary corpuscles into fibrillæ. In neither of these instances will it be doubted that the cement corpuscles themselves, or the living matter held in their territories, have in an active way taken part in the reappearance, first, of protoplasm, and afterwards of medullary corpuscles. The theory that immigrated medullary corpuscles, or "leucocytes," have replaced the former cement tissue, must be abandoned as soon as we can trace a gradual transformation of the tissue of the cementum into medullary tissue. The latter immediately assumes the characteristic features of a myxomatous or fibrous connective tissue, in connection with the pericementum.

From this point of view there is no difficulty in explaining the appearance of multinuclear bodies, so-called "myeloplaxes," in the dissolved territories. We know that such formations represent a stage of development of cementum, and they simply reappear as soon as the basis-substance of an already-formed cementum is dissolved or liquefied. In fact, nothing else is required but a re-formation of basis-substance and its recalcification, in order to reproduce new bony tissue, such as we often meet with on the periphery of absorbed cementum.

The result of the absorption is next a myxomatous or fibrous connective tissue, freely supplied with newly-formed blood vessels. In this tissue an active, new formation of bony trabeculæ of bone takes place, characterized by the presence of large and irregular bone corpuscles. The widened socket, or dissolving surface of an absorbing provisional tooth, is not infrequently filled with newly-formed bone. The newly-formed layer of cementum in part shows circular fields (territories) of bone tissue, each of which may contain a varying number of bone corpuscles, or there is a uniform reduction of the original cementum, the boundary of which is made up by regularly arranged medullary corpuscles, so-called "osteoblasts."

On the neck of the tooth the excavations penetrate not only the layer of the cementum proper, but also the layer of the subjacent dentine, which we know to be destitute of dentinal canaliculi. Here again we observe, at first a dissolution of the basis-substance in globular fields, which appear filled at first with a finely granular protoplasm, lacking nuclei, afterwards with usually nucleated medullary corpuscles, and at length with a slightly fibrillated tissue, the latter undoubtedly originating from a splitting of the medullary corpuscles into a number of delicate spindles. The surface of the neck of the tooth likewise exhibits the characteristic bay-like excavations which are filled with nucleated medullary corpuscles, or with multinuclear protoplasmic bodies. From what I have seen, I can not doubt that the nuclei, under all circumstances, are secondary formations, and can not be regarded as the future bone corpuscles. A territory of bone tissue will form only after the protoplasm has assumed a uniform granulation, and the bone corpuscles will develop out of this protoplasm by an increase of living matter at certain regular intervals. The result of the absorption and reappearance of the embryonal condition of the tissues constituting the neck of the tooth results in the formation of new bone tissue, either in the shape of globular fields (territories) of newly-formed bone tissue, or in the formation of a thin layer of regularly lamellated bone tissue, blending with that formed out of the cementum of the roots.

Bodecker, in his article on the distribution of the living matter in the dentinal tissues (Dental Cosmos, 1878–79), describes and illustrates the neck of a tooth, calling it an anomalous formation of cementum. From what I have seen, I can not doubt that his figure is taken from a deciduous tooth, exhibiting newly-formed bone tissue on the neck. Unquestionably such bony formations are not lasting, but are reformed into medullary tissue with the advancing absorption of the dental tissues, either leading to the formation of new bone, or of fibrous connective tissue.—*Trans. N. Y. State Dental Soc.*

